# Evaluatıon of Depressıon and Anxıety Status in Patıents After Cardıac Devıce Implantatıon

**DOI:** 10.1111/anec.70085

**Published:** 2025-04-25

**Authors:** Murat Özmen, Onur Altunkaya, Selim Aydemir, Sidar Şiyar Aydın, Faruk Aydınyılmaz, Emrah Aksakal, Emre Alkan

**Affiliations:** ^1^ Department of Cardiology University of Health Sciences, Erzurum Bolge Training and Research Hospital Erzurum Turkey; ^2^ Department of Cardiology Ataturk University Erzurum Turkey; ^3^ Department of Psychiatry University of Health Sciences, Erzurum Bolge Training and Research Hospital Erzurum Turkey

**Keywords:** anxiety, depression, implantable cardioverter defibrillator, pacemaker, prevalence

## Abstract

**Introduction:**

Implantable cardioverter defibrillator (ICD) and pacemaker (PM) implantation may lead to anxiety and depression, which may reduce patients' quality of life. The aim of our study was to compare depression and anxiety following device implantation.

**Methods:**

This prospective study was conducted on 202 patients admitted to our hospital with ICD and PM implants between January 2024 and January 2025.

**Results:**

The prevalence of anxiety in PM and ICD recipients was 25.9% and 38.8%, respectively. There was a significant difference in anxiety in ICD patients (< 0.001). In terms of depression, the results in the PM and ICD groups were 18.9 and 38.9, respectively. The results showed a significant difference in the prevalence of depression between PM and ICD recipients. The tendency toward depression was statistically significant in both device recipients (PM; *p* = 0.008, ICD; *p* < 0.001).

**Conclusion:**

Considering the prevalence of anxiety and depression, it seems necessary to pay closer attention to the anxiety and depression states of patients who have been treated with PM and ICD devices and to provide more widespread education to these patients.

## Graphical abstract

Although pacemaker implantation reduces mortality and morbidity in patients, the psychological state of patients may be overlooked by clinicians. Since clinicians focus on device technologies during patient check‐ups, the psychological state of the patient may be overlooked. According to our current study, patients undergoing pacemaker implantation should be provided with psychological support before and after the procedure.

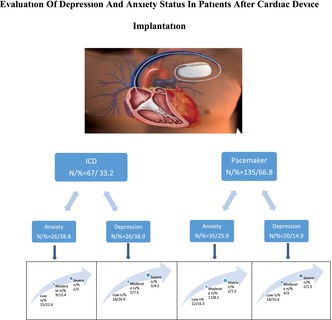

## 1 Introduction

The placement of a foreign body in the heart or a foreign substance in the body can be considered a significant event in human life (Carroll et al. 2020). In this context, PM or ICD implantation may cause an altered mental image. On the one hand, it causes problems in the body's psychosocial adaptation and quality of life, and on the other hand, it causes emotional disorders. In this context, psychological status can be affected in most cardiovascular diseases. This situation has come to the fore in many studies (Ardahanlı et al. 2021; Kaygı and Bir 2024).

Implantation of a PM or ICD has been shown to significantly improve health‐related quality of life (Lopez‐Liria et al. 2019). ICDs can be used to manage heart rhythms in general and have been shown to reduce the rate of sudden cardiac death in patients with arrhythmias (McCarthy et al. 2020). A selected patient group with appropriate indications will experience these devices as either an ICD or PM recipient. ICD shocks are painful shocks that occur in unexpected situations. However, patients do not feel PM‐induced warnings. Differences may change patients' perception of their pacemakers and their quality of life (Dougherty et al. 2020).

Physicians often need to be made aware of the seriousness of electronic devices' psychological effects on the heart. Since these devices have a complex structure, the main focus in patient controls is on the technical equipment of the device. Due to this situation, the psychosocial characteristics of the patients may be ignored (Pike et al. 2020). Considering this situation, it would be useful to understand the psychological factors that will affect patients' health and to train physicians in patient management (Lee et al. 2020). Although most ICD recipients adapt to the device, some suffer from psychological diseases. There is limited information in the literature regarding the factors affecting the psychological adaptation of patients with ICD implantation (Andersen et al. 2020).

Depression and anxiety are the most critical psychological problems in these patients. Research shows that the prevalence of depression in heart patients is approximately three times higher than in other people (24%–68%) (Hwang and Choi 2016). Depression leads to disruption of the hypothalamic–pituitary–adrenal pathway and the sympathetic system. It is an important risk factor in heart patients. Meanwhile, long‐term cortisol and norepinephrine secretion increases, and circadian order is disrupted. Depression increases cortisol levels at night. Considering the ideas put forward as the cause of death in heart disease, there are orthostatic stimulations, namely increased heart rate with standing, abnormal heart rate given to premature ventricular contractions and unusual ventricular depolarizations (Carney and Freedland 2017). Anxiety is among the serious psychological problems seen in heart disease patients (Krawczyk et al. 2020). In one cohort, more than 28% of heart disease was associated with anxiety disorders (Rao et al. 2020). Anxiety encompasses a variety of common conditions, such as depression, and is associated with depression (Peter et al. 2020).

As clinicians have focused more on the technical features of ICD and PM devices, they have paid less attention to the psychological effects of these devices on patients and their impact on their quality of life.

## 2 Material and Methods

### 2.1 Study Design

This study was conducted on 202 patients who applied to the cardiology clinic between 10 January 2024 and 1 January 2025.

### 2.2 Data Collection

Patients participating in the current study voluntarily completed the informed consent questionnaire. The participants were then given the questionnaire. They were given 25 min to complete the questionnaire. The researcher filled out questionnaires for illiterate patients through interviews. Then, questionnaires were collected from the participants. The patients were called for control 3 months after implantation.

Anxiety questionnaires consist of a two‐part questionnaire, which constitutes the data collection tools: the Beck Depression Inventory (BDI‐II)^11^ and the Beck Anxiety Inventory (BAI) (Peter et al. 2020). Demographic data included gender, age, marital status, education level, history of mental illness, use of psychiatric medication, and shock. BAI is a 21‐item inventory whose validity and reliability have been proven in many studies (α = 0.92) (Sanford et al. 2008). BDI is a standardized 21‐item scale with proven validity and reliability confirmed by many studies (α = 0.91) (Stefan‐Dabson et al. 2007). The overall score of BAI and BDI‐II is between 0 and 63. Scores of 0–7, 8–15, 16–25 and 26–63 for anxiety disorder represent no anxiety, mild, moderate and severe anxiety, respectively. Regarding depressive disorder, scores of 0–13, 14–19, 20–28 and 29–63 indicate no depression, mild, moderate and severe depression, respectively.

### 2.3 Statistical Analysis

The collected data were collected using SPSS (version 27) data analysis, descriptive (mean, percentage and frequency) and analytical (chi‐squared) tests. Student's *t*‐test was used to compare the quantitative characteristics of the patients between groups, and the chi‐squared test was used to compare the quantitative characteristics of the patients between the groups. To examine the information about the differences before and after treatment and the magnitude of the difference, the most powerful sign test, the Wilcoxon signed ranks test, was used. The Wilcoxon signed ranks test was used to compare the difference in anxiety and depression scales between the two groups (PM and ICD recipients). A *p* value of < 0.05 was considered statistically significant.

## 3 Results

PM implantation was performed in 66.8% (*n* = 135) of 202 patients, and ICD implantation was performed in 33.2% (*n* = 67). The average age was 65 ± 12 years in PM recipients and 65 ± 14 years in ICD recipients (*p* = 0.839). A total of 13 patients in both groups had a history of psychological illness, and 10 patients had a history of drug use. Demographic and clinical characteristics of the patients are shown in Table 1.

**TABLE 1:** Clinical, laboratory, and demographic data of the patients.

Group variables	Pacemaker *N* = 135/%	ICD *N* = 67/%	*p*
Age	65 ± 12	65 ± 14	0.83
Gender (male) *n*/%	99/(% 73.3)	37/(% 55.2)	**0.11**
HT *n*/%	92/(% 68)	35/(% 52.2)	0.02*
CHF *n*/%	88/(% 65)	49/(% 73.1)	0.25
CAD *n*/%	106/(% 78.5)	45/(% 67.2)	0.08
DM *n*/%	25/(% 18.5)	12/(% 17.9)	0.91
Marital status married/single *n*/%	120/(% 88.9) 15/(% 11.1)	58/(% 86.6) 9/(% 13.4)	0.63
Psychological illness *n*/%	8/(% 5.9)	5/(%7.4)	0.20
Drug use *n*/%	6/(%4.4)	4/(%5.9)	0.06
Shock *n*/%	—	7/(% 10.4)	< 0.001*
WBC 10^3^/mm^3^	12.9 ± 6.4	11.8 ± 11.1	0.003*
HGB g/Dl	14.8 ± 2.1	15.9 ± 8.6	0.65
PLT 10^3^/mm^3^	283 ± 106	266 ± 88	0.16
CRP mg/dL	30.2 ± 4.8	21.3 ± 3.5	0.17
Albumin mg/dL	43.5 ± 5.1	42.9 ± 3.7	0.07
Glucose mg/dL	127 ± 85	121 ± 78	0.02*
Total cholesterol mg/dL	167 ± 43	168 ± 42	0.74
HDL mg/dL	40 ± 10	42 ± 12	0.44
LDL mg/dL	114 ± 51	130 ± 80	0.30
Triglyceride mg/dL	149 ± 80	152 ± 87	0.96
Creatinine mg/dl	1.4 ± 1	1.1 ± 0.4	0.003*
Uric acid mg/dL	6.9 ± 2.3	6.2 ± 1.7	0.039*
Sodium mmol/L	142 ± 4.4	142 ± 2.7	0.011*
Potassium mmol/L	4.8 ± 0.6	4.6 ± 0.5	0.044*
Calcium mmol/L	9.5 ± 0.5	9.4 ± 0.4	0.64
Magnesium mmol/L	2.2 ± 0.4	2.08 ± 0.4	0.02*

Abbreviations: CHF, congestive heart failure; CAD, coronary artery disease; CRP, C‐reactive protein; DM, diabetes mellitus; HDL, high‐density lipoprotein; HGB, hemoglobin; HT, hypertension; LDL, low‐density lipoprotein; PLT, platelet; WBC, white blood cell. *= *p* < 0.05.

The overall prevalence of low‐grade anxiety in the PM and ICD groups was 16.3% and 44.8%, respectively. Regarding moderate and severe anxiety, the results showed that 9.6% and 16.4% of patients in the PM and ICD groups, respectively, experienced moderate to high anxiety. The overall prevalence of low‐grade depression in the PM and ICD groups was shown to be 13.3% and 26.9%, respectively. Regarding moderate and severe depression, the results were 4.5% and 12% of patients in the PM and ICD groups, respectively. The prevalence of anxiety and depression in PM and ICD recipients is shown in Table 2. No significant difference was observed in the prevalence of anxiety among those who received PM implants (p=0.218). Statistical significance was observed in terms of depression in PM patients (*p* < 0.001). A statistically significant difference was observed in terms of anxiety and depression in ICD recipients (*p* < 0.001; *p* < 0.001) (Table 3).

**TABLE 2:** Prevalence of anxiety and depression in PM and ICD recipients.

Group variables		Before pacemaker *N* = 135/%	After pacemaker *N* = 135/%	Before ICD *N* = 67/%	After ICD *N* = 67/%
Anxiety	No (*n*/%)	104/77	100/74.1	66/98.5	41/61.2
	Low (*n*/%)	22/16.3	22/16.3	1/1.5	15/22.4
	Moderate (*n*/%)	4/3	11/8.1	—	9/13.4
	Severe (*n*/%)	5/3.7	2/1.5	—	2/3.0
Depression	No (*n*/%)	120/88.9	115/85.1	65/97.0	41/61.1
	Low (*n*/%)	12/8.9	14/10.4	2/3.0	18/26.9
	Moderate (*n*/%)	3/2.2	4/3.0	—	5/7.5
	Severe (*n*/%)	—	2/1.5	—	3/4.5

**TABLE 3:** Comparison of depression and anxiety in PM and ICD recipients.

		Negative ranks	Positive ranks	Ties	Total	*Z* score	*p*
Pacemaker	BAI	4	7	124	135	−1.232	0.21
	BDI	2	12	121	135	−2.673	0.008
ICD	BAI	0	26	41	67	−4.594	< 0.001
	BDI	0	24	43	67	−4.517	< 0.001

*Note:* Wilcoxon signed ranks test.

Abbreviations: BAI, beck anxiety inventory; BDI, beck depression inventory.

## 4 Discussion

Although pacemaker implantation is successful in managing heart rhythm, it can cause psychological problems in patients. The current study investigated depression and anxiety in cardiac device recipients. Most of the participants in this study were men. Similar studies were conducted in Germany and the United States, and 52.7% and 52.8% of participants were male, respectively. In Zurich, of 70 participants, 50 were men and 26 were women (Brunner et al. 2004; Duru et al. 2001; Ozcan et al. 2001). The results showed that, while the increase in anxiety was not significant in PM recipients, depression was significant. A significant increase in anxiety and depression was observed among ICD recipients. While a positive increase in anxiety was observed in seven patients with PM, an increase in depression was observed in 14 patients. In ICD recipients, increased anxiety was observed in 26 patients, while depression was observed in 24 patients. Among PM recipients, the prevalence of anxiety and depression was observed to be, respectively (25.9% and 38.8%); ıt was consistent with the work of Aydemir et al. (1997), which suggested that 19% of patients suffered from depression. The prevalence of anxiety and depression was higher in ICD recipients (38.8% vs. 38.9%), meaning that people with ICDs experienced higher levels of anxiety and depression. These current findings are consistent with the findings of the studies by Webster et al. (2014). Unlike the study conducted by Thylen et al. (2014), the female population experienced more anxiety. In our study, anxiety and depression were more common in the male population. The studies by Duru, Leosdotirin and Polycandriotin were inconsistent with our current study. In the study conducted by Duru et al. (2001), the prevalence of anxiety and depression remained at a lower level. In the study conducted by Leosdottir et al. (2006), no statistical difference was observed. In the study of Polycandriotin, more anxiety and less depression were observed, especially in PM recipients (Polikandrioti et al. 2018). This observed difference may be due to differences in sample size, measurement time and method and depression anxiety scale.

The higher prevalence of depression in patients with ICD implantation may be due to increased anxiety in these individuals due to fear of shock and death. It should also be kept in mind that there may be a greater tendency for anxiety and depression after shock administration, especially in patients in the ICD group. ICD recipients often suffer from long‐term depression, and their quality of life improves over a more extended period compared to PM recipients.

Certain factors may contribute to depression after pacemaker implantation, including low physical activity, high stress, obesity, and hypertension (HT) (Dhar and Barton 2016). Consistent with our study, depression was observed to be more common in PM recipients. Focusing on activities, getting social support, getting enough rest, and developing coping strategies can improve symptoms of depression. Information and exchange of ideas, especially in patient support groups, can provide alternative perspectives on implanted devices (Rafsanjani et al. 2021). Additionally, educational interventions that emphasize the goals, functions, and positive effects of these devices can be developed and may help reduce depression (Mansouri et al. 2019). In these patients, whether they are PM or ICD users, conditions such as device malfunction, physical discomfort, battery exhaustion, and sad mood after battery implantation can trigger depression and anxiety (Figueroa et al. 2016).

As explained above, some educational interventions, especially programs that include the target, function, and positive interventions of devices, can reduce depression and anxiety.

## 5 Limitations and Strengths

One of the limitations of our study is the difficulty experienced by the participants in completing the survey and trying to solve the questions by providing explanations. Another limitation is the small sample size and being a single‐center study. Despite the current limitations, this is the first study conducted in our region. Similar studies can be conducted with a larger sample size.

## 6 Conclusıon

The results of the current study suggest that depression and anxiety are essential conditions among ICD and PM recipients. While there was no significant difference in the increase in anxiety, especially in PM recipients, the increase in depression was significant, and a significant increase in anxiety and depression was observed among ICD recipients. Considering the frequency of depression and anxiety, educating patients about the devices through booklets or brochures, informing physicians, organizing patient follow‐up programs, and organizing psychology and communication courses for physicians and healthcare personnel may be effective in reducing depression and anxiety.

## Author Contributions

All authors contributed to the work that is being presented here. M.Ö.: design, literature search and data analysis; O.A. and S.A.: manuscript preparation and manuscript editing; S.Ş.A. and F.A.: data analysis, statistical analysis; E.A. and E.A.: design and manuscript review.

## Ethics Statement

This study was approved by the Erzurum City Hospital Ethics Committee with the decision numbered 2024/01‐15. Informed written consent was obtained from all participants before the study, and they were assured that their information would be kept confidential.

## Conflicts of Interest

The authors declare no conflicts of interest.

## Data Availability

The datasets generated during and/or analyzed during the current study are available from the corresponding author on reasonable request.
